# COVID-19 Remote Learning Transition in Spring 2020: Class Structures, Student Perceptions, and Inequality in College Courses

**DOI:** 10.1177/0092055X20954263

**Published:** 2020-10

**Authors:** Alanna Gillis, Laura M. Krull

**Affiliations:** 1 St. Lawrence University, Canton, NY, USA; 2 St. Norbert College, De Pere, WI, USA

**Keywords:** COVID-19 pandemic, inequalities, online teaching, hybrid courses, crisis teaching

## Abstract

The COVID-19 pandemic forced all face-to-face college courses to transition to remote instruction. This article explores instructional techniques used in the transition, student perceptions of effectiveness/enjoyment/accessibility of those techniques, barriers that students faced due to the transition, and race/class/gender inequality in experiencing those barriers. We used surveys in introductory courses by two instructors (the authors) to compare students’ reactions to our transitions and the transitions in their other courses. We found that which instructional technique instructors use is less important than how well they implement it for student learning. Although there is a tradeoff between enjoyment and accessibility, instructors can use techniques to increase accessibility of interactive formats. Internet and technology barriers were extremely common, even for students who did not anticipate problems. Most students experienced barriers to their learning due to the pandemic, including distractions, increased anxiety, and feeling less motivated, especially for nonwhite, female, and first-generation college students.

In spring 2020, higher education experienced a huge disruption: All in-person courses transitioned to remote instruction due to the COVID-19 pandemic. Often with little to no training, instructors made rapid decisions about how to adjust their courses for remote instruction. According to early journalistic reporting, the most common strategy was to embed the existing course in a learning management system (LMS) while holding synchronous meetings; this remote transition maintained the same teaching strategies, activities, and outcomes from face-to-face learning (Lederman 2020a; Supiano 2020b). Many faculty also reported changing how students were assessed, such as by modifying exam formats or reducing the number of assignments (Lederman 2020a, 2020b). These preliminary journalistic findings also show that a small subset of faculty shifted their course to a primarily asynchronous format, allowing students to move through course content in a more flexible, self-paced way (Supiano 2020a). Students, meanwhile, had to adjust to these new course structures along with many added barriers in their own lives that made completing their academic work more difficult. Technology issues quickly surfaced: Lack of reliable Internet, a dedicated workspace, or adequate technology particularly impacted participation in synchronous meetings, such as those held over web-conferencing software like Zoom (Flaherty 2020; Lederman 2020b). Additionally, many students found themselves balancing multiple commitments, such as child care or work responsibilities, which impacted their ability to learn successfully. In analyzing the transition to emergency remote instruction, it is imperative to consider the prevalence of such barriers as well as how some groups were at disproportionate risk of encountering them, thus potentially leading to unequal learning outcomes.

Since the rapid expansion of online courses in the past few decades, scholars have identified best practices for online teaching, evaluated technologies and their place in the (virtual) classroom, and analyzed learning outcomes across diverse learning environments (Driscoll et al. 2012; Price et al. 2016; Woodley et al. 2017). Research has consistently highlighted the importance of organized course design, regular communication between faculty and students, and extensive institutional support for faculty as they design online courses (Alston 2017; Bailey and Card 2009; Martin et al. 2019). In *Teaching Sociology*, articles have analyzed general pedagogical strategies and early challenges in online teaching (Benson et al. 2002; Jaffee 2003), how to enhance the classroom experience using online tools (Belet 2018; Persell 2004; Wyant and Bowen 2018), and student outcomes and satisfaction in online versus face-to-face classes (Driscoll et al. 2012). Although such research is helpful for contextualizing and evaluating student experiences of the emergency transition to remote learning in the spring of 2020, we recognize that this transition, although heavily reliant on technologies commonly found in online teaching, represents a distinct teaching and learning phenomenon.

Given that instructors are currently trying to plan for an uncertain 2020–2021 academic year and that future natural disasters and crises may potentially disrupt face-to-face learning, we need rigorous analysis of the spring 2020 transition from the instructors’ *and* the students’ perspectives. Several surveys of faculty have been published in *Inside Higher Ed* and *Chronicle of Higher Education*, but we would do well to supplement these early observations with analysis of students’ responses to different instructional methods. Knowing what strategies for online learning were perceived as effective, enjoyable, and accessible from the students’ view will be important for faculty designing flexible courses for fall 2020 and beyond. In this article, we use data collected throughout our own transitions to online teaching to analyze students’ perceptions of the emergency transition, evaluate the usefulness of various online learning strategies, and analyze barriers encountered.

## Literature Review

Because little research has been published thus far on the spring 2020 emergency transition, our literature review explores prior research on best practices in online teaching, synchronous compared to asynchronous approaches, and barriers to online teaching and learning. Based on this review, we then analyze how this past research on online teaching may apply to the distinct phenomenon of emergency remote teaching as experienced in spring 2020.

### Best Practices in Online Teaching

#### Know the students

Teaching in any context benefits from knowing the types of students likely to enroll, including their motivations for taking the course and their demographics (Bricknell and Muldoon 2012). Research comparing online to face-to-face students finds that students who self-select into online courses are likely to have lower GPAs and to work more hours in their jobs (Driscoll et al. 2012). Additionally, students in online courses are not necessarily technologically literate (Fish and Wickersham 2009), so faculty may consider implementing ungraded assessments that give students practice engaging with relevant online technology (Woodley et al. 2017). In the event of an emergency remote transition, students’ needs and challenges have likely changed, and instructors may want to take the time to familiarize themselves with their students’ emerging concerns, questions, and situations.

#### Start with learning objectives

As with any teaching, the course must begin with clearly articulated learning objectives (Martin et al. 2019). Once instructors create course learning objectives, they can develop specific activities that align with at least one of those learning objectives (Alston 2017; Martin et al. 2019). This approach can encourage instructors to use only technology that will help students meet the learning objectives rather than using new digital tools simply because they are flashy (Bailey and Card 2009; Clark-Ibáñez and Scott 2008; Woodley et al. 2017). Finally, instructors should recognize that teaching online is not simply a matter of moving existing materials online. Serious thought should be given to the presentation of content, the types of activities included, and more (Bricknell and Muldoon 2012; Keengwe and Kidd 2010). Of course, in the context of a remote transition, this careful alignment between learning objectives and course material may be more difficult to achieve given the need to move quickly and to potentially learn new instructional technologies.

#### Select appropriate tools

Once learning objectives are established, instructors can evaluate different instructional tools with these objectives in mind. For example, an instructor whose learning objectives include “develop critical thinking and analytical skills” may hope to partially fulfill that objective via student-to-student discussion. However, they could accomplish that asynchronously via discussion forums or synchronously via small group conversations on Zoom or a similar platform. Instructors would then want to consider both their own and their students’ familiarity with a given form of technology as well as how to implement best practices. For example, discussion forums are most effective at fostering student learning and engagement when the instructor provides clear, structured guidelines and prompts (Andreson 2009; Clark-Ibáñez and Scott 2008; Hsiao 2010; Martin et al. 2019; Persell 2004). Instructors should first detail their requirements for how to participate in the forums and provide rubrics for how students will be assessed (Clark-Ibáñez and Scott 2008; Martin et al. 2019). Then, they should develop structured—rather than open-ended or free-response—prompts that tend to promote higher-level thinking; instructors could assign roles to students (Parrott and Cherry 2011), such as a starter who explains his or her key takeaway from the reading and a responder who answers questions raised by the starter (Persell 2004), or they might write prompts asking students to connect concepts to their own experiences (Andreson 2009; Clark-Ibáñez and Scott 2008). An instructor considering a synchronous requirement to foster critical thinking—such as having groups of three to four students meet via Zoom to discuss course content—may want to provide groups flexibility to schedule their own meeting times (Hsiao 2010). Research shows that an opportunity for synchronous interaction enhances student integration and learning in fully online classes, but requiring frequent synchronous interactions risks creating barriers for students with technology, time, and resource constraints (Hsiao 2010; Skylar 2009; Woodley et al. 2017).

#### Clear organization and expectations

One of the best ways to ensure student learning, engagement, and enjoyment in online courses is to have clear organization (Bailey and Card 2009; Martin et al. 2019). Content should be presented in manageable amounts and in consistent ways; for example, if a course is organized using learning modules, then each module should have the same appearance and information, such as readings, due dates, and assignments (Alston 2017; Bricknell and Muldoon 2012; Martin et al. 2019). Furthermore, students should know from the outset how they are expected to participate online (Price et al. 2016). When faculty communicate high expectations, they encourage students to commit to their learning (Bailey and Card 2009). In the context of a remote transition, new organization strategies need to be developed quickly and consistently.

#### Prompt and regular communication

In an online learning environment, faculty must regularly access the course page, respond consistently to student inquiries, and grade in a timely manner (Alston 2017; Bailey and Card 2009). In online learning, timeliness is more critical for student success than facilitating synchronous interactions (Martin et al. 2019). Additionally, students appreciate an instructor’s engagement with the course (Price et al. 2016); higher levels of instructor interaction predict improved learning and higher course satisfaction (Boling et al. 2012; Driscoll et al. 2012; Fish and Wickersham 2009). In an emergency remote transition, this regular communication can also serve to reduce student anxiety about the transition.

### Synchronous Versus Asynchronous Approaches

The emergency transition to online learning sparked a debate about the relative merits of a synchronous, asynchronous, or blended approach to online instruction. Synchronous classes require students and faculty to be online at the same time, over a virtual platform such as Zoom, which promotes interaction but restrains flexibility (Hsiao 2010; Skylar 2009). Additionally, it requires instructors and students to have access to the necessary technology and workspace at a specific time, which may be difficult if multiple people in a household are working remotely or if students have other time-specific responsibilities. Asynchronous classes are characterized by greater flexibility and more options for self-paced learning (Hsiao 2019). However, significant drawbacks include less interaction or excessive reading and writing assignments (Boling et al. 2012). A blended approach seeks a balance of these two strategies, perhaps organizing one synchronous session a week and otherwise using discussion forums.

Although the debate about synchronous versus asynchronous approaches proliferated in 2020, most existing research explores asynchronous class formats or analyzes the effect of adding synchronous elements to primarily asynchronous courses (Skylar 2009). For example, Hsiao (2010) analyzed students’ experiences of asynchronous and synchronous interactions and found that students appreciated the flexibility of asynchronous forums but appreciated the occasional opportunity to engage in real time with others. Boling et al. (2012) found that from the student perspective, the most successful online classes incorporated diverse strategies for engagement, such as live conversations with experts in their field. However, there was not a comparison online course that relied on regularly scheduled classes; instead, synchronous meetings were one of many tools that instructors used.

### Barriers to Online Teaching and Learning

Start hereOf longstanding concern is the digital divide, which referred first to unequal access to technology and later to how people engage differently with digital resources (Belet 2018; Benson et al. 2002; Hargittai 2003). Access to computer and Internet technology has always been stratified, with racial and ethnic minorities, people from lower socioeconomic backgrounds, and people in rural areas being less likely to have a computer and to connect to the Internet (Clark and Gorski 2002; Hargittai 2003). Unequal access can contribute to lower rates of digital literacy, which can put students at a disadvantage in educational settings (Hargittai 2003). Early research during the COVID-19 pandemic showed such disadvantages emerging within months of schools transitioning to remote instruction (Vogels et al. 2020). Additionally, issues with Internet connectivity can make engaging in synchronous virtual discussions even more challenging, and unanticipated loss of Internet could be disruptive in asynchronous contexts, such as when completing an online exam (Olt and Teman 2018; Ryabov 2012).

Such barriers may be exacerbated and new ones may emerge when learning in a crisis such as the COVID-19 pandemic. For example, in normal times, students with Internet connectivity issues at home may work in the campus library or coffee shops; however, this option is not possible when everything is shut down. Additionally, with many members of a household potentially working from home, Internet slowdowns can occur, making synchronous classes more difficult for some students to attend. Faculty, too, may lack sufficient Internet connectivity or technology resources to work from home. Finally, anxiety is heightened; during COVID-19, students and faculty are concerned not just about technology issues but also health, financial (in)stability, and safety at home. Students may be working extra hours to support themselves and/or their family, taking on additional caregiving responsibilities at home, or experiencing uncertainty around their living situations (e.g., if they were unable to return home when campuses closed), among other concerns. The high levels of stress and anxiety that result from these barriers may make schoolwork difficult to prioritize, especially for low-income students and students of color who are even more likely to face these challenges.

### Applying Existing Findings to Emergency Remote Teaching

The speed necessitated by an emergency transition may make some best practices in online teaching particularly salient while rendering others less applicable. Due to the likelihood of future emergency transitions of face-to-face courses in fall 2020, this section reviews how emergency remote teaching may differ from the previous research on online teaching. First, in an emergency transition, neither the students nor the instructor chose an online class, meaning there may be steep learning curves for everyone. Neither students nor faculty likely anticipate having to finish their courses online, so they may not have the necessary technology, and individuals who do have that technology may still need instruction in how to use it. Second, instructors may have to modify their learning objectives and reimagine their course to successfully achieve those objectives via online learning. In doing so, instructors should recognize that stressors caused by the COVID-19 pandemic—or other events that could trigger extended remote transitions such as local natural disasters—may negatively impact student engagement in coursework. Additionally, common online assignments, such as real-world observations, may no longer be possible in a pandemic or other emergency, and instructors must consider the ability of students to safely complete any projects. For instance, previous research done following Hurricane Katrina in New Orleans demonstrated that the emergency remote conditions challenged both students and faculty as they quickly learned new technology and adapted to the chaotic conditions (Angelocci, Lacho, and Bradley 2008). The mental health impacts of such a natural disaster, as evidenced by high rates of posttraumatic stress disorder (PTSD) after Hurricane Katrina, may pose a barrier to educational success (Phillips and Phillips 2008). Experts are similarly worried about high rates of PTSD and other mental health concerns due to the COVID-19 pandemic (Xiao, Luo, and Xiao 2020).

Third, clear organization and communication may be particularly critical during an emergency transition or another crisis. By organizing online content in a consistent manner and ensuring students understand what is (newly) expected of them, faculty may be able to more successfully facilitate the transition from face-to-face to online learning. Explaining how students can succeed in the online portion of the class may help alleviate stress and increase students’ learning. Fourth, students often raise the concern of lacking interpersonal connection with the instructor and with other students in online courses (Hsiao 2010). This issue may be somewhat mitigated if students had opportunities to collaborate prior to the emergency transition, as with group assignments or structured small group discussions, and if instructors maintain such learning strategies when moving their courses online. However, for classes that made an emergency transition immediately before classes began (i.e., fourth-quarter classes for universities on quarter systems or schools that initially planned to be in person for fall 2020 but pivoted to entirely remote instruction with little advance notice), students may be even more concerned about experiencing interpersonal connection. Thus, professors may need to address this concern directly and intentionally from the beginning of the course.

By systematically analyzing students’ perceptions of the spring 2020 emergency transition to remote instruction, we can assess not only the effectiveness and accessibility of diverse instructional tools but also the barriers that students encountered that impeded their learning, which could prove helpful in future emergency transitions. Next, we turn our attention to how we transitioned our courses prior to discussing our methods and data.

## Our Adjustments for Remote Learning

Gillis had no previous experience in online teaching, whereas Krull had taught four sections of Introduction to Sociology (IntroSoc) online. In the spring of 2020, we were both teaching face-to-face IntroSoc at the same institution. Due to differences in goals as we moved our courses online, we used some overlapping and some distinct instructional techniques. Gillis’s goals were to continue dynamic peer-to-peer discussions, make the coursework as accessible as possible, and keep students as engaged as possible. Accordingly, I created a new assignment category called lesson plans, where students received a completion grade for writing summaries of their experience with the day’s activity and their responses to discussion questions. I used each of three lesson plan formats approximately once a week: small group video discussions, where students met in their groups within a 48-hour window to discuss questions and submit collective answers; forums, where students had 48 hours to answer a set of questions and then respond to at least two group members; and individual worksheets. However, students could complete any lesson plan with the small group video format if they preferred. Students did not have to tell me in advance when they would be meeting, and I did not drop in on the discussions with their groups unless they requested I join their discussion. I had assigned students to groups of four at the beginning of the semester, and by keeping these same groups, I hoped that students would remain engaged. Based on student feedback, I added PowerPoints in VoiceThread to each lesson plan instead of sending out my discussion lecture notes; I also added two live Zoom discussion classes, but I gave them the option to complete those days’ lesson plans on their own. Some of these changes relate to best practices outlined previously regarding flexibility in scheduling meeting times and having alternatives to synchronous meetings for those who cannot attend (Hsiao 2010) as well as always structuring the remote class with synchronous meetings (Skylar 2009; Woodley et al. 2017).

Krull completed the transition to remote instruction with three goals in mind: to facilitate student learning while minimizing anxiety connected to the course, maintain student/student and instructor/student interactions, and maintain consistency. To achieve the first goal, Krull switched to an asynchronous format using our institution’s LMS, which enabled more self-paced learning and created flexibility for student engagement. Knowing the importance of consistency and clear organization, I kept deadlines for weekly assessments such as quizzes and forum posts, and I communicated deadlines each week via email and via the course syllabus (Bailey and Card 2009; Martin et al. 2019). However, I granted extensions when students asked, frequently reminding them of this possibility. To maintain interactions, I created structured discussion forums using the groups that students had been assigned prior to remote instruction. For each class, students were either a discussion starter or a responder, reflecting the recommended practice of assigning roles for forum discussions, and prompts generally required students to write their own discussion questions, role play, and/or analyze data I provided (Parrott and Cherry 2011; Persell 2004). I held virtual drop-in office hours during our regularly scheduled class time to connect with students, and I met with nearly half of my students at least once. To maintain consistency, I kept all of my planned assignments, but I changed due dates and modified the requirements for several. Additionally, once I decided to use an asynchronous approach and to rely heavily on forums, I committed to this strategy for the remaining five weeks of the semester.

**Table 1.** Summary of Transition to Remote Instruction for Each Course.

Goal	Original Approach	Pandemic Approach
Gillis		
Dynamic peer-to-peer discussions	Assigned small groups for class discussions and activities	Created daily “lesson plans” that typically required small group video meetings or forum postings
Accessible coursework	Clear assignment guidelines with flexible deadlines Multidimensional participation grade, with grade largely based on student improvement along self-created goals (Gillis 2019)	Flexibility to complete small group meetings and forum postings within a 48-hour window Always had option to submit an individual assignment instead of working with group Students adjusted participation goals for remote coursework
Maintain student engagement	Active learning lesson plans involving small group and full class discussions Occasional mini-lectures of ~5 minutes to introduce key concepts	Used same PowerPoint structure to present discussion questions and activities in lesson plan Created short VoiceThread presentation to recap takeaways from discussion questions and provide mini-lectures, per student requests Led two discussion-based live Zoom classes, per student requests
Krull		
Facilitate learning while minimizing anxiety	Granted extensions when asked Consistent deadlines for daily quizzes	Granted extensions when asked and frequently reminded students to ask Consistent deadlines for quizzes, forum post, and forum replies
Encourage faculty/student and student/student interactions	Office hours in my office two days a week and by appointment Small group work each class period with assigned groups Group presentation during final exam period	Office hours on Zoom during class time and by appointment Forum discussions with same prepandemic group members and assigned roles Group presentation created and viewed asynchronously
Maintain consistency with prepandemic expectations and with pandemic expectations	Project that requires original photos Three-page paper that should use six concepts or resources from class	Project that requires any photos, as long as properly cited Two- to three-page paper that should use three concepts or resources from class Used same forum approach for duration of semester

## Methods and Data

To analyze our transitions, we use several surveys collected at different time points in our IntroSoc courses. Gillis sent three surveys: one in mid-March during the extended spring break, one after the first week of remote instruction, and one the last week of class (see timeline in Table 2). Students were given a completion quiz grade for completing each survey. Krull had two surveys: one in mid-March and one during the week of final exams. The students were not given an incentive for completing the surveys, but they were strongly encouraged to do so. The first surveys were designed separately to solicit feedback from our students about the emergency transition, and we initially created them as a way to learn more about students’ initial concerns and living situations at the start of the online transition. However, when Gillis realized these surveys could also be used for research, she invited Krull to use the same instrument for the final survey; thus, with the exception of a few questions, the final survey is identical across both courses and is the basis for most analysis in this article. The surveys primarily asked close-ended questions, with a few open-ended ones. In this article, we analyze responses to the following: “How EFFECTIVE were each of the following types of lesson plans for your LEARNING in [this class/other classes]?” “How ENJOYABLE were each of the following types of lesson plans for you personally in [this class/other classes]?” and “How ACCESSIBLE were each of the following types of lesson plans for you in [this class/other classes], given the constraints you faced this last month?” Answer choices included very, somewhat, and not. To analyze barriers, we primarily used the question, “To what extent did each of the following impact your ability to succeed academically [in this class/other classes]? Answer choices were to a great extent, to a limited extent, not at all, and not applicable.

**Table 2.** Timeline of Remote Learning and Research Methodology.

March 9–15	March 16–22	March 23–29	March 30–April 5	April 6–19	April 20–26	April 27–30
Spring break College announces transition to remote learning and an additional week of spring break	Second week of spring break First surveys for Gillis and Krull	Remote learning begins	Gillis second survey Gillis changes based on survey	Remote learning continues	Last week of remote learning Gillis students sign consent forms Gillis students final survey	Final exam week Krull students sign consent forms Krull students final survey

At the end of the semester, both instructors asked students to complete consent forms to allow their student records to be used in research, per Institutional Review Board instructions. In Gillis’s course, students uploaded the consent form as an assignment on the LMS, although whether students checked yes or no was not seen until final grades were submitted. In Krull’s course, there was no incentive to complete the consent form, although they were strongly encouraged to do so. As a result, the courses had different participation rates. Gillis had 40 of 44 (91 percent) students sign the consent form; of the 40 with consent forms, 39 (98 percent) students completed all three survey waves, and all 40 completed the final survey. Krull had 29 of 48 (60 percent) students sign the consent form; of the 29 with consent forms, 20 (69 percent) completed the first survey, and 26 (90 percent) completed the second survey. Thus, there is likely a selection effect, especially in Krull’s course; we recognize that our results here likely underestimate the prevalence of barriers experienced by students.

Our methods and data have many strengths. First, we provide some of the first systematic analysis of how the remote transition impacted teaching and learning using student-centered data. Students reported their perceptions of the effectiveness,^1^ enjoyment, and accessibility of various instructional techniques and the extent to which different barriers impacted their educational success. A second strength of these data is that we have several waves of responses on a few measures; for example, we analyze anticipated to actual Internet problems.^2^ A third strength is the timing of the surveys: The first surveys were sent out within five days of the university announcing the change to remote learning, thus enabling us to capture much of the uncertainty students felt. Additionally, the surveys were timely. When students were asked to complete the final survey, they were still finishing their coursework, and so the barriers they faced were fresh on their minds. A final strength is that the surveys were distributed in two courses and additionally asked about students’ other courses, enabling multiple points of comparison to analyze students’ experiences with diverse instructional techniques. In addition to our IntroSoc courses, 27 percent of students took at least one other sociology course; 50 percent took at least one nonsociology, social science course; 77 percent took at least one STEM course; and 66 percent took at least one arts/humanities course.

Nevertheless, there were also several limitations to this study. First, because the first surveys were not designed for research, the questions from these are not fully aligned. Second, the list of instructional techniques (small group video chat, small group forum, individual worksheet/lesson plan, live Zoom class discussion, live Zoom class lecture, VoiceThread PowerPoint, and drop-in office hours) is based on what we used and what we heard anecdotally about other classes; they should not be treated as an exhaustive list. A third limitation is that we asked students to report on their experience in IntroSoc and “other courses,” losing some of the distinctions between other courses. For instance, students may have thought forums were effective in one course but ineffective in another, and that variation is not captured. A fourth limitation is that the total sample size of 66 students resulted in many demographic variables needing to be binary, such as white and nonwhite and first-generation college student and non-first-generation college student. We acknowledge that important differences exist within these categories and hope future research with larger sample sizes can address them. Finally, the sample used here is not representative of all college students, nor is our institution generalizable to all college institutions. Our institution is an elite, residential, public research university in the southeast. Our sample was 70 percent women, 68 percent white, and 71 percent non-first-generation college student, whereas the undergraduate student body as a whole is 60 percent women, 59 percent white, and 81 percent non-first generation, meaning that our sample contains more women, white students, and first-generation students. Furthermore, the student population and instructional techniques analyzed here are likely not representative of all colleges or college students. Nevertheless, we believe many of the lessons learned here can be helpful to most institutions of higher education.

## Results and Discussion

Next, we review the results of our surveys. Because our findings lend themselves to immediate discussion, we incorporate more discussion in the results than one might typically find in an article. We believe this approach increases clarity for readers and helps identify practical takeaways from the findings.

### Instructional Techniques

Instructors used various techniques during the remote portion of spring 2020 semester, as seen in Table 3. These categories are not mutually exclusive given that instructors possibly used a combination of strategies. Both synchronous and asynchronous techniques were common: 92 percent of students had at least one class that met live, and the most common instructional techniques reported were live drop-in office hours and lecture-based live Zoom classes. However, of the students who had at least one class meet live, 16 percent reported having a class that did not meet at the originally scheduled time, one of many barriers to synchronous courses that we will discuss in the following.

**Table 3.** Percentage of Students Reporting at Least One Course, Other Than Ours, Using This Technique during the Remote Transition.

Instructional Technique	Percentage of Students
Drop-in office hours^a^	88.5
Zoom: lecture based	87.9
VoiceThread	78.8
Individual worksheet/lesson plan	75.8
Zoom: discussion based	69.7
Forum	60.6
Small group video chat	59.1

a This technique was only included in the survey for Krull’s course. All other techniques were included in the surveys for both courses.

Although all instructional techniques were fairly common, they were not equally effective, enjoyable, or accessible. Table 4 shows the percentage of students who rated each technique as very effective, enjoyable, or accessible for their IntroSoc course and their other courses.

**Table 4.** Percentage of Students Reporting Instructional Technique Being Very Effective, Enjoyable, and Accessible by Course.

Instructional Technique	Other Course (*N* = 66 Total^a^)	Gillis (*N* = 40)	Krull (*N* = 26)
Drop-in office hours (*N* = 23)			
Effectiveness	56.5	—	57.7 (16)
Enjoyment	38.1	—	53.9 (18)
Accessibility	50	—	84.6 (23)
Live Zoom lecture (*N* = 58)			
Effectiveness	55.2	—	—
Enjoyment	36.2	—	—
Accessibility	71.7	—	—
VoiceThread (*N* = 52)			
Effectiveness	50	67.5	42.3 (25)
Enjoyment	28.0	37.5	46.2 (26)
Accessibility	80.8	87.5	84.6 (26)
Individual worksheets/lesson plan (*N* = 50)			
Effectiveness	30	57.5	—
Enjoyment	14.3	20.0	—
Accessibility	94.2	95.0	—
Live Zoom discussion (*N* = 46)			
Effectiveness	41.3	70	—
Enjoyment	31.3	72.5	—
Accessibility	77.6	70.0	—
Forums (*N* = 40)			
Effectiveness	22.5	17.5	61.5
Enjoyment	15.4	5.0	46.2
Accessibility	88.4	95.0	88.4
Small group video chat (*N* = 39)			
Effectiveness	33.3	77.5	—
Enjoyment	23.7	67.5	—
Accessibility	70.7	82.5	—

a The sample size varies because if a student reported that none of their other classes used that technique, they were not included in the calculation. For Gillis, all percentages correspond to a sample size of 40. For Krull, students were given the option of not applicable for VoiceThread and drop-in office hours, and so the sample size for each question’s response is shown in parentheses.

Student perception of effectiveness varied considerably across courses. For example, the effectiveness of forums ranged from 18 percent for Gillis, to 23 percent for other classes, to 62 percent for Krull. The open-ended questions further reiterate the differences. For Gillis, in response to a question about what the instructor should not do again, a student wrote, “Forum posts, I do not feel that they significantly aided in group discussions and building upon ideas. They were more of just an assignment we completed rather than small group discussions to actually talk and connect.” This response was not unusual: 15 out of 36 valid open-ended responses to this question exclusively talked about forums for Gillis. In contrast, Krull’s students were generally content with the forums and included them in the responses about what the instructor should do again. For example, one student said,

I think the forum posts were effective in replacing the peer discussion. I don’t think anything could match live peer discussion, but the forum posts provided a place for me to think about the material. . . . It made me understand the material better writing about it. We could also see our peers’ response, so I could still understand different perspectives.

Fourteen of the 25 valid open-ended responses about what to keep for Krull mentioned the forums.

These differences may have arisen for two reasons. First, Krull had experience with online teaching, including forums, so she incorporated best practices for forum discussions, including assigning students to roles and creating effective prompts. In contrast, Gillis had no forum experience and simply posted the same questions she typically used for small group discussions in face-to-face learning. Based on student comments, Gillis’s structure resulted in students feeling that forum posts were individual assignments made more difficult because they had to respond to classmates. Another possible explanation for the difference in reported effectiveness is that Gillis’s students experienced a variety of techniques, all of which they rated higher than forums, whereas forums were the main component of Krull’s remote course. Thus, Gillis’s students may have felt discontent due to more points of comparison.

Another technique that varied in effectiveness was live Zoom discussions: 70 percent of Gillis’s students rated this as very effective, yet only 41 percent did so for other courses. The open-ended responses again suggest possible reasons. The first reason is that Gillis structured the live Zoom discussions similarly to her face-to-face classes, including moving between small group and full class discussions. As a student stated, “The live zoom classes worked . . . almost as good as being in class.” Another reason may be that Zoom discussions were occasional rather than every class period, allowing students to benefit from the technique without being overly burdened by it. For example, one student explained that Gillis should continue to “Not hold live zoom meeting each class period” if she were to teach remotely again. Another commented, “You gave us choices and polled us on lesson formats. Most just continued with synchronous lectures which for me was sometimes difficult (like 10:30 p.m. classes).” Thus, live Zoom classes can be effective, but they are not automatically so. Overall, these results suggest that the effectiveness of instructional techniques during this remote transition was less about which techniques were used than how each technique was implemented within the course.

Our results also reveal a tradeoff between synchronous interaction, enjoyment, and accessibility. Asynchronous techniques such as forums, individual worksheets, and VoiceThread were rated by almost all students as very accessible. However, these techniques tended to be less enjoyable; none of them were rated as very enjoyable by even half the students. Student enjoyment is important, given that online courses have higher attrition rates, and when students enjoy their courses, they are more likely to continue engaging (Darby and Lang 2019). Nevertheless, these data suggest that instructors can successfully balance accessibility and enjoyment, such as by offering alternatives to synchronous classes. For instance, 70 percent of Gillis’s students rated live Zoom discussions as very accessible, and 83 percent of students rated small group video as very accessible. The presence of alternative lesson plans for students who could not attend live Zoom sessions as well as students’ ability to schedule their own meeting time for small group discussion likely contributed to these high accessibility ratings. Indeed, students frequently expressed appreciation for these flexible options. One student wrote: “Thankful that we get to organize our own time to meet (thank you!) because that has made it much easier.” This flexibility in meeting time may help explain why the same technique of small group video chat was more likely to be rated as very accessible in Gillis’s course compared to other courses; how a technique is implemented can impact its accessibility. By not always requiring synchronous course meetings and by granting students autonomy in scheduling small group meetings, Gillis successfully balanced interaction, enjoyment, and accessibility, from the students’ perspective.

### Barriers

Nevertheless, many barriers did impact students’ success in their courses, from their perspective. A major barrier was Internet and technology access. As seen in Table 5, more than half of students, 20 out of 39, encountered at least occasional Internet problems during the five weeks of remote instruction, with 8 percent of students experiencing these problems often. All students who predicted having problems did have them, but only 58 percent of students who predicted never having problems were correct in that prediction.^3^ Ultimately, many more students had Internet and technology problems than anticipated, something that instructors need to take into serious consideration when creating course policies. We imagine that students attending lower resourced or more rural universities may have even more Internet problems than our students experienced.

**Table 5.** Percentage of Actual Internet Problems by Predicted^a^ Internet Problems in Any Course.

Actual Internet Problems	Predicted Never	Predicted Occasionally	Predicted Unsure	Total *N*
Never	58.1	0.0	33.3	19
Occasionally	38.7	80.0	33.3	17
Often	3.2	20.0	33.3	3
Total *N*	31.0	5.0	3.0	39

a Students had the choice to predict often/no Internet access, but no student chose that option, so it is omitted from the table. Data only include students from Gillis.

Many other barriers impacted student success. For instance, even at this elite university, students experienced housing insecurity. Gillis’s first survey revealed that 15 percent of students were not sure where they would be living during the remote portion of the course. We also analyzed students’ experiences of barriers related to their living situation, anxiety and stress around COVID-19, and academic or course changes. Table 6 shows the percentage of students reporting if each barrier impacted their academic success in our courses and their other courses. The barriers due to their new living situation were nearly universal, especially the distraction of being in a new workspace. These barriers were roughly the same across courses, demonstrating that instructors had little ability to mitigate the effect of these barriers. Likewise, barriers directly connected to COVID-19 were similarly and commonly experienced across courses. Most students felt their academic success was inhibited by feeling unmotivated, distracted, and/or anxious due to COVID-19. The majority also reported feeling less motivated due to mental health concerns and having trouble sleeping. Less commonly, although for more than one in four students, they also felt their academics suffered due to worrying about personal finances and accessing medical care.

**Table 6.** Frequency of Each Barrier Inhibiting Academic Success by Course.

Barrier	Combined Gillis and Krull (*N* = 66)	Other Courses (*N* = 66)
Barriers due to new living situation		
Distraction: new workspace	92.4	95.4
Privacy of new living space	68.2	72.7
No dedicated workspace	65.1	71.2
Barriers directly due to COVID-19		
Unmotivated: COVID-19	81.8	78.8
Distraction: COVID-19	77.3	78.8
Anxious: COVID-19	69.7	69.7
Unmotivated: mental health	56.1	59.1
Having troubles sleeping	53.0	51.5
Worried: personal finances	39.4	37.9
Worried: access medical care	25.8	25.8
Barriers due to academic or course changes		
Unmotivated: not in person	86.4	87.9
Fewer opportunities for peer discussions	69.7	80.3
Fewer opportunities for questions	53.0	72.7
Unmotivated: pass/fail option	30.3	51.5
Insufficient flexibility of coursework	28.8	69.7

*Note:* Due to selection effects into the survey for Krull, our two courses are not analyzed separately. However, comparisons between combined SOCI 101 courses and other courses are valid because the same students responded to each question. We can thus analyze differences in course policies between our courses and others.

Nevertheless, course structure can shape the relative impact of some of the barriers related to academic changes. Almost all students felt less motivation due to not meeting in person. However, our courses had fewer students reporting lower motivation due to pass/fail option, fewer opportunities for questions, fewer opportunities for peer discussions, and insufficient flexibility of coursework. Although many students still encountered these barriers, these differences show that instructors can reduce the number of students who experience barriers. In open-ended responses, we both received comments from students about our willingness to adapt the course to student needs. One student stated, “Thank you for asking for our input and genuinely caring how we’re doing with this transition. Sadly, you’re the only professor of mine who has asked for input and seems to want to help us and do the best remote teaching possible.” It is generally a good practice to solicit feedback from students, but it is especially important to do so when student needs are evolving. For instance, Gillis’s students used the survey to request video clips of mini-lectures. In response, she incorporated VoiceThread, and as shown previously, students generally found this very effective for their learning.

We also both incorporated flexibility while maintaining structure, which students reported helped minimize potential barriers. For instance, a student of Gillis wrote, “I appreciate the leniency in your deadlines because it gave me time to get things done, but the fact that there were deadlines held me accountable.” Similarly, a student of Krull wrote,

By having assignments and readings with forum posts due regularly, you kept me up to date with the information I needed to learn. Other classes simply posted youtube videos or powerpoints and expected students to do them regularly on their own. As a result, in other classes I have fallen behind on the information.

Students need flexibility on deadlines and lesson plans as various barriers arise, but they still need structure to help hold them accountable for their learning.

Another practice that seemed to help reduce academic barriers for students was our incorporation of structured opportunities for connecting with peers. As one student from Krull’s stated, “I really liked that we were placed in groups because I really liked being able to talk about the different things that we were learning in a small group setting.” In contrast, a different student in Krull’s course said this was a major barrier in other courses when they were unable to continue meaningful peer-to-peer interactions: “The major thing that I found I am missing is the interaction with my peers to ask questions and clarify topics. Especially in my STEM class, it is helpful to sit next to a friend so we can discuss material in the moment if we have questions.” Such meaningful peer-to-peer learning opportunities may get lost in the emergency transition to remote instruction, especially if instructors do not create structured opportunities for group work.

A final strategy that students identified as helpful for reducing academic barriers was maintaining clear communication, such as by answering questions quickly. Student comments suggest we created an online learning environment in which they could easily ask us questions without fear of judgment. They specifically cited drop-in office hours and detailed class communications. For example, one of Krull’s students wrote: “I really liked having office hours over Zoom during remote learning. I think it incentivized me in all my classes to go to office hours more to ask questions because it was so much more convenient.” Krull also created an “online learning” syllabus in Google Docs as a means of centralizing communication related to the course. One student commented, “The new syllabus as a google document with all the links included was very helpful in staying organized for assignments.” Many students expressed difficulty in keeping up with course-related emails, and so this strategy allowed information to be centrally shared rather than distributed across multiple emails. In general, students benefitted from having one centralized place in which they could find all course information, be it a Google Docs syllabus or in a section of the LMS.

Finally, we found that not all students were equally likely to experience each of these barriers. Table 7 shows the percentage of students who experienced each of the barriers in our courses broken down by race, class, and gender.^4^ Although not all differences were statistically significant, often due to the small sample size, there seems to be a suggested correlation between these demographic variables and some barriers. For instance, nonwhite students were more likely to feel unmotivated due to COVID-19, suffer from less flexibility of coursework (significant), be worried about their finances (significant), and be worried about accessing medical care (significant). First-generation college students were more likely to lack a dedicated workspace (significant), suffer from less flexibility of coursework, and be worried about finances. Women were more likely to lack a dedicated workspace (significant), be worried about personal finances, and be worried about accessing medical care.

**Table 7.** Frequency of Selected Barriers Inhibiting Academic Success by Race, Class, and Gender of Student.

Barrier	Nonwhite (*N* = 21)	White (*N* = 45)	Male (*N* = 20)	Female (*N* = 46)	Non-First-Generation (*N* = 46)	First-Generation (*N* = 19)
No dedicated workspace	66.7	64.4	45.0*	73.9*	56.5*	84.2*
Unmotivated: COVID-19	95.2	75.6	75.0	84.8	80.4	84.2
Worried: personal finances	57.1*	31.1*	25.0	45.7	37	52.6
Worried: access medical care	42.9*	17.8*	10.0	32.6	26.1	26.3
Insufficient flexibility of coursework	47.6*	20.0*	30.0	28.3	19.6	47.4

* χ^2^ test significant at .05.

The open-ended responses identified additional barriers that we did not include in the close-ended survey questions. For instance, students mentioned being in a different time zone, not having access to the textbook that was left in their inaccessible dorm room, struggling to communicate with group members for projects, and getting a new job with hours that conflicted with course meeting times, among others. Thus, the aforementioned list should not be taken as an exhaustive list of all barriers that students faced but instead can be a guide for thinking through the kinds of barriers students might face; how those barriers might be differently impactful based on race, class, and gender; and how much control instructors have to reduce those barriers in their classrooms. Furthermore, the frequency of these barriers reflects the fact that our institution is an elite, residential university. Students who attend other kinds of institutions such as comprehensive, regional, or community colleges likely experienced even more barriers, such as lacking child care, even greater housing instability, greater work conflicts, and greater financial instability (McMurtrie 2020).

## Conclusion

Our article makes four important contributions to the scholarship of teaching and learning. First, this article provides a critical first look at how students perceived remote learning during an emergency transition. Teaching during a global pandemic makes even clearer the importance of communication and accessibility as well as the need to be aware of barriers (both anticipated and unanticipated) that may arise to negatively impact students’ learning. For example, technology problems are particularly detrimental when students cannot seek out other solutions outside the house and most technology stores are closed. Nevertheless, we should extend this concern to all teaching contexts because even in the best of times, some people may have inconsistent Internet access or may be unable to work in public spaces with Internet. Thus, even when students opt to take online courses and acquire the necessary technology, they may still encounter unanticipated technological difficulties. Faculty would do well to consider course policies that may help mitigate unequal learning due to technological issues outside of the students’ control.

Second, the effectiveness, accessibility, and enjoyability of diverse instructional strategies and digital tools can vary considerably depending on how each strategy is implemented. Simply picking one strategy that would seem to be enjoyable and interactive—such as Zoom—does not automatically mean that students will perceive that strategy to be effective, accessible, or enjoyable. Faculty must also be intentional in how they implement strategies in support of their learning goals. A critical component for this success is training because simply knowing how the technology works does not necessarily ensure successful learning. For instance, Gillis knew how to use the LMS to create forum discussions, but without knowledge of how to successfully design forums for student learning, her forums were ineffective.^5^ Furthermore, by creating multiple check-ins for students, faculty can learn what is working well and what is not during the semester, identifying barriers to accessibility and effectiveness as they arise and then addressing them promptly. For example, Gillis successfully adjusted part of her content delivery when students asked for recorded mini-lectures as opposed to only notes. In contrast, whereas Krull worked with students one-on-one when elements of the course needed modifications or had become inaccessible, in the future, she would implement more check-ins to make it easier for students to raise any concerns. Advance planning is key to success, but that planning does not preclude the necessity of flexibility as needs and circumstances change. Furthermore, when considering what online tools and instructional technologies to use, instructors may want to consider not just their priorities regarding accessibility, enjoyment, and effectiveness but also their own knowledge of and comfort with such tools. Selecting one or two techniques that align with their course goals and employing them effectively may ultimately be more successful than using many or all of the strategies analyzed here.

Third, our findings complicate the synchronous versus asynchronous debates that emerged in spring of 2020. Students do value interaction and recognize the importance of both student/student and faculty/student interactions for their learning, but they did not universally praise Zoom above asynchronous tools. Live Zoom lectures (which neither of us used) had the second highest effectiveness rating (55 percent) for techniques in other courses, but enjoyment and accessibility were on par with other instructional strategies. Although not a perfect comparison, Krull’s forums had higher numbers for all three categories, suggesting that when forums are implemented well and tap into higher-level thinking skills, students feel engaged with the course and with each other. Future research on online learning strategies may want to continue evaluating and analyzing how strategies are implemented rather than focusing primarily on comparing between strategies (Hsiao 2010; Skylar 2009). For example, what Zoom norms contribute to more effective learning in live lectures?

Finally, our results show high levels of anxiety, distraction, and lack of motivation for all students, although students from underresourced backgrounds are disproportionately likely to encounter other barriers to learning, such as lack of a dedicated workspace or worry about finances. COVID-19-specific barriers are likely to persist in the foreseeable future, so faculty would do well to consider what practice and policies they can implement to mitigate these barriers. Creating flexible course options, instructing students in using technology, setting clear but manageable expectations, and maintaining open lines of communication will set students and faculty up for success not only in the uncertain 2020–2021 academic year but in any situation where uncertainty and anxiety create more barriers to learning.

In all, this study provides a starting point for analyzing students’ perceptions of their courses during the emergency remote transition of spring 2020. Nevertheless, the data were gathered primarily as feedback for us as instructors, with varying survey questions and incentives for participation. More rigorous research methods with larger sample sizes will be needed to further analyze many of these issues at hand. Likewise, our study examines data exclusively from an elite flagship research university. We would expect many of our findings to hold in broad strokes, such as students having widespread and unanticipated technology problems and facing academic barriers directly and indirectly related to COVID-19 and students from marginalized backgrounds disproportionately expe-riencing these challenges, given that these findings align with previous research from teaching and learning and sociology of education. However, how students perceive the effectiveness, enjoyability, and accessibility may differ more at other kinds of institutions. For instance, only one of our students had a child at home. Although others cared for siblings, our largely traditionally aged college students may have been more likely to enjoy the synchronous interpersonal connections with peers, whereas students at community colleges and regional universities with more family obligations may be more likely to find enjoyable coursework that they are able to complete asynchronously at a time that fits their schedules. Only future research at diverse institutions with diverse student populations can address these questions of critical importance to ensure that instructors develop instructional techniques that fit the needs of the students in their classrooms.

## Notes

1. Measuring student perception of effectiveness is an indirect assessment of learning, not a direct one (Sweet and Cardwell 2016). However, due to our data limitations, it is the best measure we can use in this analysis.

2. Although many students had some Internet problems and several had major Internet problems, no students were completely without Internet access for the entire semester. In Krull’s class, students who did not complete a survey or the consent form successfully engaged in forums and daily quizzes, suggesting that Internet access alone was likely not a major problem contributing to the lower response rate.

3. The same analysis was run for predicted technology issues (e.g., computer or tablet not working) compared to actual technology problems, and the results were similar. Results available on request.

4. We include a few selected barriers due to space constraints, although all barriers analyzed by race, class, and gender are available on request.

5. Two strategies Krull found to be successful were requiring students to develop discussion questions themselves and using a simplified, asynchronous roleplay. First, to help students write strong discussion questions, I provided several examples and a clear rubric. I similarly modeled different quality responses by writing examples of my own. Second, I modified several TRAILS role-playing resources (Ferguson, Andercheck, and McClure 2015; Gillis 2018) to fit a forum format. By requiring students to answer from a particular perspective (i.e., a disgruntled parent or a frustrated AP teacher, in Gillis’s role-play activity), students had to apply what they learned in a creative way, and they had to respond to each other in character. Resources I created for forums are available on request
